# Association of State Supplemental Nutrition Assistance Program Eligibility Policies With Adult Mental Health and Suicidality

**DOI:** 10.1001/jamanetworkopen.2023.8415

**Published:** 2023-04-14

**Authors:** Anna E. Austin, Madeline Frank, Meghan E. Shanahan, H. Luz McNaughton Reyes, Giselle Corbie, Rebecca B. Naumann

**Affiliations:** 1 Department of Maternal and Child Health, Gillings School of Global Public Health, University of North Carolina at Chapel Hill, Chapel Hill; 2 Injury Prevention Research Center, University of North Carolina at Chapel Hill, Chapel Hill; 3 School of Social Work, University of North Carolina at Chapel Hill, Chapel Hill; 4 Department of Health Behavior, Gillings School of Global Public Health, University of North Carolina at Chapel Hill, Chapel Hill; 5 Center for Health Equity Research, Department School of Medicine, University of North Carolina at Chapel Hill, Chapel Hill; 6 Department of Epidemiology, Gillings School of Global Public Health, University of North Carolina at Chapel Hill, Chapel Hill

## Abstract

**Importance:**

Food insecurity is associated with an increased likelihood of poor mental health and suicidality. The Supplemental Nutrition Assistance Program (SNAP) is the largest program addressing food insecurity in the US; under broad-based categorical eligibility (BBCE), states have the option to expand SNAP eligibility to a greater number of households by eliminating the asset test or increasing the income limit for eligibility.

**Objectives:**

To examine the association of state elimination of the asset test and increases in the income limit for SNAP eligibility with rates of mental health and suicidality outcomes among adults.

**Design, Setting, and Participants:**

This ecological cross-sectional study used 2014 to 2017 data on US adults from the National Vital Statistics System and 2015 to 2019 data on US adults from the National Survey on Drug Use and Health (NSDUH) State-Level Small Area Estimates. Analyses were conducted between September and November 2022.

**Exposures:**

State elimination of the asset test only and state adoption of both SNAP eligibility policies (ie, state elimination of the asset test and increases in the income limit) for 2014 to 2017 from the SNAP Policy Database.

**Main Outcomes and Measures:**

Number of adults with a past-year major depressive disorder, mental illness, serious mental illness, or suicidal ideation and number of adults who died by suicide.

**Results:**

Analyses included 407 391 adult NSDUH participants and 173 085 adults who died by suicide. State elimination of the asset test only was associated with decreased rates of past-year major depressive episodes (rate ratio [RR], 0.92; 95% CI, 0.87-0.98) and mental illness (RR, 0.91; 95% CI, 0.87-0.97) among adults. State adoption of both SNAP eligibility policies (ie, state elimination of the asset test and increases in the income limit) was associated with decreased rates of past-year major depressive episodes (RR, 0.92; 95% CI, 0.86-0.99), mental illness (RR, 0.92; 95% CI, 0.87-0.98), serious mental illness (RR, 0.91; 95% CI, 0.84-0.99), and suicidal ideation (RR, 0.89; 95% CI, 0.82-0.96). Results suggested a decreased rate of suicide death (RR, 0.93; 95% CI, 0.84-1.02) in states with both policies compared with states with neither policy, although this result was not statistically significant.

**Conclusions and Relevance:**

State adoption of policies that expand SNAP eligibility may contribute to decreased rates of multiple mental health and suicidality outcomes at the population level.

## Key Points

**Question:**

Is state adoption of policies that expand Supplemental Nutrition Assistance Program (SNAP) eligibility by eliminating the asset test and increasing the income limit associated with changes in rates of mental health and suicidality outcomes among adults?

**Findings:**

In this ecological cross-sectional study, state elimination of the asset test only was associated with decreased rates of past-year major depressive episodes and mental illness among adults. State adoption of both policies (ie, elimination of the asset test and increases in the income limit) was associated with decreased rates of past-year major depressive episodes, mental illness, serious mental illness, suicidal ideation, and suicide death among adults.

**Meaning:**

These results suggest that state adoption of policies that increase the number of households eligible to receive food purchasing assistance through SNAP may contribute to reductions in rates of poor mental health and suicidality among adults at the population level.

## Teaser

This cross-sectional study examines the association of 2 policies that allow greater access to Supplemental Nutrition Assistance Program (SNAP) benefits with reductions in mental health issues and suicide.

## Introduction

Mental health disorders and suicidality contribute substantially to morbidity and mortality in the US.^1,2^ More than 51 million US adults, representing 21% of the population, have a diagnosable mental health disorder.^2^ Each year, nearly 12 million US adults have serious thoughts of suicide, more than 3 million make suicide plans, and 1.4 million attempt suicide.^2^ The multiple social and economic stressors of the COVID-19 pandemic exacerbated an existing mental health crisis,^3,4^ with 1 in 5 US adults reporting that the pandemic significantly and negatively impacted their mental health.^5^ The ongoing mental health crisis underscores a critical need for programmatic and policy strategies that address underlying risk factors at the population level.

Food insecurity, defined as limited or uncertain access to adequate food due to lack of money or other resources,^6^ is consistently linked to poor mental health and suicidality.^7^ In the US, approximately 1 in 10 households including nearly 25 million adults experience food insecurity each year.^8^ Multiple studies show that experiencing food insecurity is associated with an increased likelihood of psychological distress,^9,10^ depression and anxiety,^11,12,13,14^ severe mental illness,^15^ suicidal ideation,^11,13^ and death by suicide.^16^ Uncertainty about the ability to access or afford enough food, as well as potentially forgoing other basic needs to purchase food, may result in feelings of anxiety, stress, guilt, and shame that negatively impact mental health and increase risk for suicidality.^17,18^

The Supplemental Nutrition Assistance Program (SNAP) is the largest program addressing food insecurity in the US. SNAP currently provides more than 21 million low-income households, including more than 41 million people, with a monthly benefit to assist with the cost of food.^19^ Households are eligible for SNAP if household income does not exceed 130% of the federal poverty level (FPL) and household assets do not exceed $2250, with some modifications for households with an older adult or person with a disability.^20^ While baseline eligibility rules are set at the federal level, states have the option to adopt policies that expand SNAP eligibility. A key policy states can adopt is broad-based categorical eligibility (BBCE).^21^ Under BBCE, states have the option to increase the income limit for SNAP eligibility to up to 200% FPL and to eliminate the asset test used to determine SNAP eligibility.^21^ Prior research shows that state adoption of these policies is associated with an increase in the number of households enrolled in SNAP^22,23,24^ and decreases in state-level poverty and food insecurity.^25,26,27^ By expanding and stabilizing food supports for a larger percentage of the population, it is possible that state adoption of these policies may contribute to improvements in mental health at the population-level. Prior research demonstrates benefits of other state-level policies that aim to improve household economic conditions, including more generous state Earned Income Tax Credits (EITC)^28,29^ and higher state minimum wage,^30,31,32^ for mental health at the population level.

The aims of this study were to examine the association of state elimination of the asset test and state increases in the income limit for SNAP eligibility under BBCE with rates of major depressive episodes, mental illness, serious mental illness, suicidal ideation, and suicide deaths among adults. Given that food insecurity is associated with an increased likelihood of poor mental health and suicidality, and state elimination of the asset test and increases in the income limit for SNAP eligibility are associated with decreases in food insecurity, we hypothesized that state adoption of these policies would be associated with decreased rates of poor mental health and suicidality outcomes.

## Methods

This study was considered exempt by the institutional review board at the University of North Carolina at Chapel Hill and did not require informed consent because data were deidentified. It followed the Strengthening the Reporting of Observational Studies in Epidemiology (STROBE) reporting guideline.

### Data Sources

We used data from the SNAP Policy Database, the National Survey on Drug Use and Health (NSDUH) State-Level Small Area Estimates, and the National Vital Statistics System (NVSS). The SNAP Policy Database is available online from the US Department of Agriculture (USDA)^33^ and provides data on state adoption of various SNAP policies through 2016. We updated data on state adoption of BBCE policies using the SNAP State Options Reports^34^ and communication with USDA and state SNAP agencies. Each year, the NSDUH interviews a representative sample of the civilian, noninstitutionalized population ages 12 years or older with questions assessing substance use, mental health, and other health-related outcomes over the past year. The NSDUH uses a state-based multistage area probability sampling design to support creation of state-level estimates.^35^ The Substance Abuse and Mental Health Services Administration (SAMHSA) uses NSDUH data to generate 2-year state-level estimates of the total number of adults ages 18 years or older with key substance use and mental health outcomes, with overlapping 2-year averages used to maintain statistical fidelity and ensure participant confidentiality. Additional details are published elsewhere.^35^ NVSS data are available online and provide data on causes of death for US residents based on death certificates.^36^

We used NSDUH state-level estimates for 2015-2016, 2016-2017, 2017-2018, and 2018-2019, the most recent years of data available. Because all data are retrospectively reported in NSDUH, we created a 1-year lag between state adoption of the SNAP eligibility policies and the first year of the 2-year state-level averages for our outcomes. For example, we considered the 2015-2016 NSDUH state estimates to align with SNAP eligibility policies in 2014 (eTable 1 in Supplement 1). Thus, we examined SNAP eligibility policies for 2014 to 2017. This approach is consistent with prior research.^29^ Because deaths in NVSS are reported in the year they occurred and are not retrospectively reported like data from NSDUH, we examined deaths that occurred in the same year as the SNAP eligibility policies. To maintain consistency with years examined for outcomes from NSDUH, we examined deaths and SNAP eligibility policies for 2014 to 2017.

### Measures

**Figure 1.**
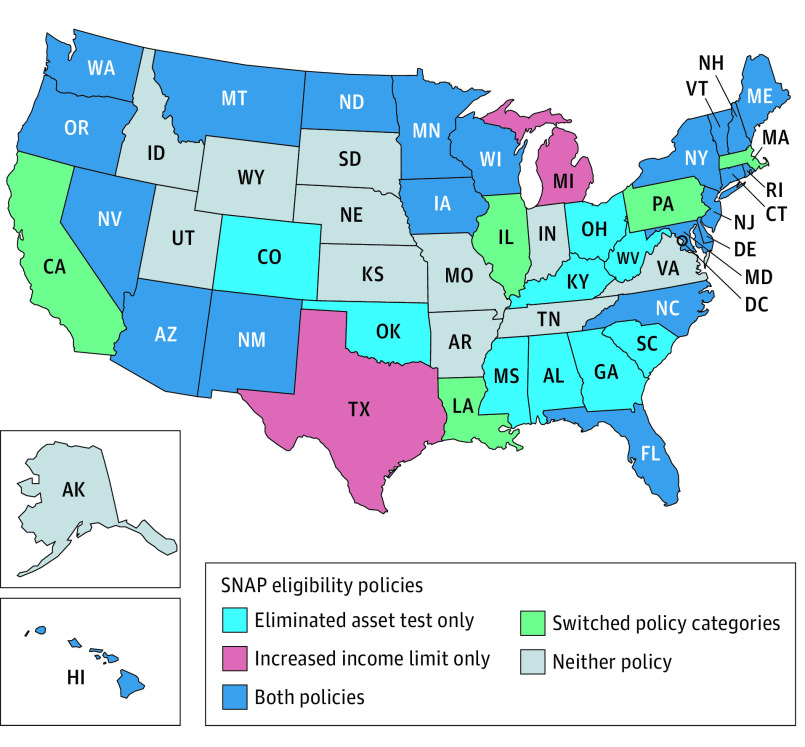
State Adoption of Supplemental Nutrition Assistance Program (SNAP) Eligibility Policies, 2014-2017 Switched policy categories indicates states that changed from neither policy to adoption of 1 or both policies between 2014 and 2017, for example (eTable 1 in Supplement 1). State-years with an increased income limit only were excluded from primary analyses due to the small number (9 state-years [4.4%]).

Our exposures were time-varying measures of state elimination of the asset test for SNAP eligibility, state increases in the income limit for SNAP eligibility, and state adoption of both policies under BBCE. We determined the year states had each policy for 2014 to 2017 from our updated SNAP Policy Database. We categorized state-years as having eliminated the asset test only, increased the income limit only, or adopted both policies (Figure 1; eTable 2 in Supplement 1). Because of the small number and percentage of state-years with an increased income limit only (9 state-years [4.4%]), we excluded these state-years from primary analyses. Thus, in primary analyses, we compared states that eliminated the asset test and states that adopted both policies (ie, eliminated the asset test and increased the income limit) with states that did not adopt either policy. Results including the 9 state-years with an increased income limit only are available in the Supplement (eFigure 1, eTable 3 in Supplement 1).

Our outcomes from the NSDUH state-level estimates were time-varying measures of the number of adults ages 18 years or older with a past-year major depressive episode, mental illness, serious mental illness, and suicidal ideation by state and year. In NSDUH, a major depressive episode in the past year is defined using the *Diagnostic and Statistical Manual of Mental Disorders* (Fourth Edition) (*DSM-IV*) criteria. Any mental illness is based on the presence of a diagnoseable mental, behavioral, or emotional disorder, regardless of the level of impairment, in the past year. Serious mental illness is based on the presence of a diagnoseable mental, behavioral, or emotional disorder resulting in substantial impairment in carrying out major life activities in the past year.^37^ Suicidal ideation is defined as an affirmative response to the question, “At any time in the past 12 months did you seriously think about trying to kill yourself?” Our outcome from NVSS was the number of suicide deaths, defined as a death with an underlying cause of death code in X60-X64, Y87.0, or U03,^38^ among adults ages 18 years or older by state and year.

We created a conceptual diagram, based on existing empirical evidence and our subject matter expertise, of variables likely to be operative in the association of state adoption of SNAP eligibility policies and our outcomes (eFigure 2 in Supplement 1). Based on our conceptual diagram, we adjusted analyses for time-varying measures by state and year of the underlying state policy context (state minimum wage, refundable EITC, maximum Temporary Assistance for Needy Families [TANF] benefit for a family of 3, Medicaid expansion, and recreational marijuana legalization) and state economic conditions (percentage of population unemployed and median household income). We did not adjust analyses for the percentage of the population by race and ethnicity as, based on our conceptual diagram, this potential confounding pathways was blocked by adjusting for state economic conditions. We obtained these data from the University of Kentucky National Welfare Database^39^ and the US Census Bureau Small Area Income and Poverty Estimates.^40^

### Statistical Analyses

To examine associations of state elimination of the asset test and adoption of both SNAP eligibility policies with the mental health and suicidality outcomes, we conducted generalized linear regression with a log link and Poisson distribution. In separate models, we used the number of adults with past-year depressive episodes, mental illness, serious mental illness, and suicidal ideation and the number of adults who died by suicide as the outcome and compared these outcomes for states that eliminated the asset test and adopted both SNAP eligibility policies to states that did not adopt either policy. In all models, we included the natural log of the population ages 18 years or older as an offset term to account for differences in population size by state and year and to calculate rate ratios (RRs) and corresponding 95% CIs. We used generalized estimating equations with an exchangeable working correlation matrix to account for repeated measures within states over time. We adjusted all models for the above noted confounders and included a linear time trend to account for secular trends over time. We conducted sensitivity analyses that also adjusted for the number of primary care physicians and the number of psychiatrists per 100 000 population,^41^ measures of the mental health workforce that may affect mental health outcomes but are unlikely to affect state adoption of SNAP eligibility policies.

To assess the potential for unmeasured confounding to affect results, we conducted analyses using the number of unintentional motor vehicle deaths among adults ages 18 years or older per 100 000 population as the outcome. We did not expect this outcome to be affected by state adoption of SNAP eligibility policies.

We conducted analyses in SAS version 9.4 (SAS Institute Inc) in September through November 2022. To interpret results, we relied on both the magnitude of RRs and the width and location of corresponding 95% CIs, in accordance with guidance from the American Statistical Association.^42^

## Results

From 2014 to 2017, the asset test was eliminated in 42 state-years (21.5%), the asset test was eliminated and the income limit was increased (ie, both policies were adopted) in 102 state-years (52.3%), and neither policy was adopted in 51 state-years (26.2%) (Figure 1). The mental health and suicidality outcomes from the 2015 to 2019 NSDUH State-Level Small Area Estimates included data from 407 391 adult NSDUH participants. Suicide deaths, identified from NVSS, included 173 085 adults who died by suicide from 2014 to 2017.

From 2014 to 2017, the percentage of adults with past-year major depressive episodes, any mental illness, serious mental illness, and suicidal ideation and the number of suicide deaths among adults per 100 000 population increased somewhat in states that had the asset test eliminated, both policies, or neither policy (eFigures 3-7 in Supplement 1). Overall, the median (IQR) percentage of adults with a past-year major depressive episode (both policies, 7.3% [6.7%-8.1%] vs neither policy, 7.7% [7.1%-8.2%]), mental illness (19.1% [18.0%-20.6%] vs 20.3% [18.8%-21.5%]), serious mental illness (4.6% [4.2%-5.2%] vs 5.2% [4.6%-5.4%]), and suicidal ideation (4.5% [4.0%-4.9%] vs 4.7% [4.4%-5.2%]) as well as the median number of suicide deaths per 100 000 adult population (18.5 [13.8-22.9] deaths per 100 000 population vs 22.6 [19.5-26.9] deaths per 100 000 population) were slightly lower in state-years with both policies compared with state-years with neither policy (eTable 4 in Supplement 1). State-years with both policies also had a lower percentage of adults with these mental health outcomes than state-years with only the asset test eliminated (eg, any mental illness: 19.1% [18.0%-20.6%] vs 19.9% [18.3%-21.5%]; suicide deaths: 18.5 [13.8-22.9] deaths per 100 000 population vs 19.5 [17.3-23.5] deaths per 100 000 population).

**Figure 2.**
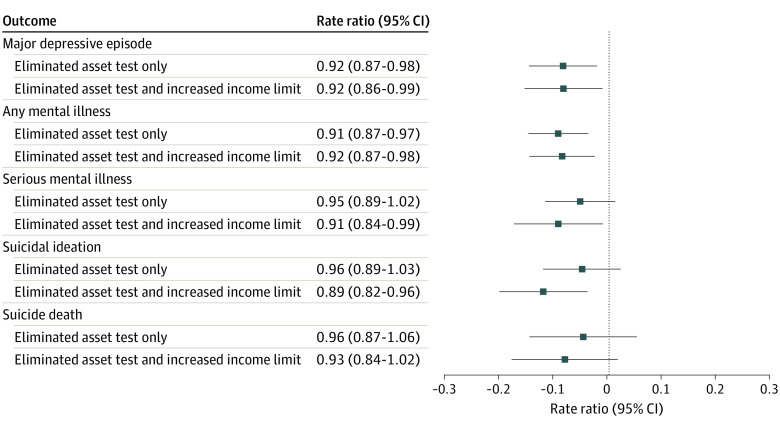
Associations of State Supplemental Nutrition Assistance Program Eligibility Policies With Mental Health Outcomes and Suicidality Among Adults^a^ Comparison group is state-years that did not have the asset test eliminated or the income limit increased for Supplemental Nutrition Assistance Program eligibility (n = 195 state-years). ^a^Adjusted for a linear time trend and state minimum wage, refundable Earned Income Tax Credit, maximum Temporary Assistance for Needy Families benefit for a family of 3, Medicaid expansion, recreational marijuana legalization, percent population unemployed, and median household income.

Adjusting for state minimum wage, refundable EITC, maximum TANF benefit for a family of 3, Medicaid expansion, recreational marijuana legalization, percentage population unemployed, median household income, and a linear time trend, state elimination of the asset test and adoption of both policies were associated with decreases in several mental health and suicidality outcomes (Figure 2, Table; confounder point estimates in eTables 5-9 in Supplement 1). State elimination of the asset test and adoption of both policies were associated with a decreased rate of past-year major depressive episodes (asset test: RR, 0.92; 95% CI, 0.87-0.98; both policies: RR, 0.92; 95% CI, 0.86-0.99) and mental illness (asset test: RR, 0.91; 95% CI, 0.87-0.97; both policies: RR, 0.92, 95% CI, 0.87-0.98) compared with state adoption of neither policy. State adoption of both policies was associated with a decreased rate of past-year serious mental illness (RR, 0.91; 95% CI, 0.84-0.99) and suicidal ideation (RR, 0.89; 95% CI, 0.82-0.96) compared with state adoption of neither policy. Results suggested a decreased rate of suicide death (RR, 0.93; 95% CI, 0.84-1.02) in states with both policies compared with states with neither policy. Although this result was not statistically significant, the location of the 95% CI and magnitude of the RR were consistent with a decreased rate of suicide death. State elimination of the asset test only was not associated with a change in the rate of past-year serious mental illness (RR, 0.95; 95% CI, 0.89-1.02), suicidal ideation (RR, 0.96; 95% CI, 0.89-1.03), or suicide death (RR, 0.96; 95% CI, 0.87-1.06) compared with state adoption of neither policy.

**Table.** Associations of State Supplemental Nutrition Assistance Program (SNAP) Eligibility Policies With Mental Health Outcomes and Suicidality Among Adults

Outcome	Rate ratio (95% CI)	
	Unadjusted	Adjusted^a^
**Major depressive episode**		
Neither policy	1 [Reference]	1 [Reference]
Eliminated the asset test only	0.87 (0.80-0.94)	0.92 (0.87-0.98)
Eliminated the asset test and increased the income limit	0.93 (0.87-0.98)	0.92 (0.86-0.99)
**Any mental illness**		
Neither policy	1 [Reference]	1 [Reference]
Eliminated the asset test only	0.88 (0.83-0.94)	0.91(0.87-0.97)
Eliminated the asset test and increased the income limit	0.94 (0.90-0.99)	0.92 (0.87-0.98)
**Serious mental illness**		
Neither policy	1 [Reference]	1 [Reference]
Eliminated the asset test only	0.87 (0.78-0.96)	0.95 (0.89-1.02)
Eliminated the asset test and increased the income limit	0.91 (0.85-0.97)	0.91 (0.84-0.99)
**Suicidal ideation**		
Neither policy	1 [Reference]	1 [Reference]
Eliminated the asset test only	0.91 (0.84-0.99)	0.96 (0.89-1.03)
Eliminated the asset test and increased the income limit	0.92 (0.86-1.00)	0.89 (0.82-0.96)
**Suicide deaths**		
Neither policy	1 [Reference]	1 [Reference]
Eliminated the asset test only	0.90 (0.82-1.00)	0.96 (0.87-1.06)
Eliminated the asset test and increased the income limit	0.90 (0.81-1.00)	0.93 (0.84-1.02)

^a^ Adjusted for a linear time trend and state minimum wage, refundable Earned Income Tax Credit, maximum Temporary Assistance for Needy Families benefit for a family of 3, Medicaid expansion, recreational marijuana legalization, percent population unemployed, and median household income (195 state-years).

Results were similar in sensitivity analyses that also adjusted for the number of primary care physicians and psychiatrists per 100 000 population (eFigure 8, eTable 10 in Supplement 1). There was no association of state elimination of the asset test only or adoption of both policies with rates of unintentional motor vehicle deaths among adults (eFigure 9, eTable 11 in Supplement 1).

## Discussion

Results indicate that state adoption of policies that expand SNAP eligibility by eliminating the asset test and increasing the income limit are associated with reduced rates of multiple mental health and suicidality outcomes at the population level. We found that state elimination of the asset test only and state elimination of the asset test and increases in the income limit (ie, state adoption of both policies) under BBCE were associated with decreased rates of major depressive episodes and mental illness among adults. We also found that state adoption of both policies was associated with decreased rates of serious mental illness and suicidal ideation. In addition, we found that states with both policies had a decreased rate of suicide death compared with states with neither policy, although this result was not statistically significant. Taken together, this suggests that multiple supportive social and economic policies may be needed to reduce more severe and less prevalent mental health and suicidality outcomes, including serious mental illness, suicidal ideation, and suicide death, at the population level.

Existing research indicates that SNAP participation contributes to improvements in mental health and that decreases in SNAP benefits adversely affect mental health.^14,27,43,44^ By eliminating the asset test and increasing the income limit for SNAP eligibility, states increase the number of households eligible for and participating in SNAP.^22,23,24^ Under these policies, there is also greater stability in SNAP benefits for participating households. For example, when states increase the income limit, household members can accept a new job with higher wages without fear of losing SNAP benefits. When states eliminate the asset test, households experiencing a sudden job and income loss do not have to spend down money in savings to become eligible for SNAP. By increasing the number of households eligible to receive food purchasing assistance through SNAP and increasing the stability of this assistance for participating households, these policies address food insecurity, a salient risk factor for poor mental health and suicidality, among a larger proportion of the population, potentially contributing to reductions in poor mental health and suicidality at the population level.

Collectively, our results and those from prior research indicate a consistent association of expanded SNAP eligibility and participation with improved mental health outcomes. While our study was ecological and conducted at the state level, prior research conducted at the individual level demonstrates associations of SNAP participation and various SNAP policies with improved mental health outcomes,^14,27,43,44^ corroborating our state-level results. State elimination of the asset test and increases in the income limit for SNAP eligibility under BBCE may be particularly important policies, as prior research shows that fluctuations in income or assets that trigger SNAP benefit reductions are associated with worse health outcomes.^27^

### Limitations

This study had several limitations. First, mental health and suicidal ideation are sensitive and stigmatized topics and thus may be underreported in NSDUH. NSDUH uses computer-assisted interviews to facilitate privacy and disclosure. However, even with potential underreporting, it is unlikely that underreporting would differ by state SNAP policy context and thus systematically bias results. Second, there is potential for misclassification of suicide deaths, with some classified as unintentional or of undetermined intent on the death certificate. Again, while this may lead to an undercount of the true number of suicide deaths, it is unlikely that this misclassification would differ by state SNAP policy context. Third, due to constraints of the data, our study population included all adults and was not limited to those eligible for SNAP. Prior research examining the association of state Medicaid expansion with suicide deaths found that expansion was associated with reduced rates in the total population but that this reduction was larger among adults with less than a college education, a population more likely to be eligible for and benefit from expansion.^45^ Fourth, although we adjusted multivariable analyses for a comprehensive set of potential confounders, there may be unmeasured confounding. To examine the potential for unmeasured confounding to bias results, we conducted analyses using unintentional motor vehicle deaths among adults as a negative control, finding no association of state adoption of SNAP eligibility policies with this outcome. Fifth, this study was ecological and thus was subject to the ecological fallacy. The results do not allow for casual inferences regarding the potential impact of SNAP participation on mental health and suicidality at the individual level.

## Conclusions

Persistently high and increasing rates of mental health disorders and suicidality in the US indicate an urgent need for population-level prevention. SNAP is a critical social safety net program addressing food insecurity, a risk factor for poor mental health and suicidality. Our results add to a growing body of evidence that social and economic support policies may be an important component of efforts to improve mental health at the population level.^28,29,30,31,32^ These results are timely given the impact of the COVID-19 pandemic on mental health^3,4,5^ and food insecurity,^46^ as well as the end of temporary SNAP benefit increases and suspended work requirements for SNAP eligibility in many states with the end of the COVID-19 public health emergency in March 2023.^47^

## Notes

Supplement 1. **eTable 1.** Alignment of Data From the National Survey on Drug Use and Health (NSDUH) With Years of State Supplemental Nutrition Assistance (SNAP) Eligibility Policies **eTable 2.** State Policy Adoption Status for Supplemental Nutrition Assistance Program (SNAP) Eligibility Policies, 2014-2017 **eFigure 1.** Associations of State Supplemental Nutrition Assistance Program (SNAP) Eligibility Policies With Past Year Mental Health Outcomes and Suicidality Among Adults Ages ≥18 Years **eTable 3.** Associations of State Supplemental Nutrition Assistance Program (SNAP) Eligibility Policies With Past Year Mental Health Outcomes and Suicidality Among Adults Ages ≥18 Years **eFigure 2.** Conceptual Diagram of Variables Likely to Be Operative in the Association Between State Adoption of Supplemental Nutrition Assistance (SNAP) Eligibility Policies and Mental Health and Suicidality Outcomes **eFigure 3.** Median Percentage of Adults Ages ≥18 Years With Past Year Major Depressive Episodes by Year and State Adoption of Supplemental Nutrition Assistance (SNAP) Eligibility Policies **eFigure 4.** Median Percentage of Adults Ages ≥18 Years With Past Year Mental Illness by Year and State Adoption of Supplemental Nutrition Assistance (SNAP) Eligibility Policies **eFigure 5.** Median Percentage of Adults Ages ≥18 Years With Past Year Serious Mental Illness by Year and State Adoption of Supplemental Nutrition Assistance (SNAP) Eligibility Policies **eFigure 6.** Median Percentage of Adults Ages ≥18 Years With Past Year Suicidal Ideation by Year and State Adoption of Supplemental Nutrition Assistance (SNAP) Eligibility Policies **eFigure 7.** Median Rate of Suicide Deaths Among Adults Ages ≥18 Years per 100 000 Population by Year and State Adoption of Supplemental Nutrition Assistance (SNAP) Eligibility Policies **eTable 4.** Adults Ages ≥18 Years With Past Year Mental Health Outcomes and Suicidality by State Adoption of Supplemental Nutrition Assistance (SNAP) Eligibility Policies **eTable 5.** Associations of State Supplemental Nutrition Assistance Program (SNAP) Eligibility Policies With Past Year Major Depressive Episode Among Adults **eTable 6.** Associations of State Supplemental Nutrition Assistance Program (SNAP) Eligibility Policies With Past Year Mental Illness Among Adults **eTable 7.** Associations of State Supplemental Nutrition Assistance Program (SNAP) Eligibility Policies With Suicide Deaths Among Adults **eTable 8.** Associations of State Supplemental Nutrition Assistance Program (SNAP) Eligibility Policies With Past Year Suicidal Ideation Among Adults **eTable 9.** Associations of State Supplemental Nutrition Assistance Program (SNAP) Eligibility Policies With Past Year Suicidal Ideation Among Adults **eFigure 8.** Associations of State Supplemental Nutrition Assistance Program (SNAP) Eligibility Policies With Past Year Mental Health Symptoms and Disorders Among Adults Ages ≥18 Years Additionally Adjusted for Measures of the Mental Health Care Workforce **eTable 10.** Associations of State Supplemental Nutrition Assistance Program (SNAP) Eligibility Policies With Past Year Mental Health Symptoms and Disorders Among Adults Ages ≥18 Years Additionally Adjusted for Measures of the Mental Health Care Workforce **eFigure 9.** Associations of State Supplemental Nutrition Assistance Program (SNAP) Eligibility Policies With Unintentional Motor Vehicle Deaths Among Adults Ages ≥18 Years **eTable 11.** Associations of State Supplemental Nutrition Assistance Program (SNAP) Eligibility Policies With Unintentional Motor Vehicle Deaths Among Adults Ages ≥18 Years

Supplement 2.
Data Sharing Statement
