# Supplementary material for: Association of State Supplemental Nutrition Assistance Program Eligibility Policies With Adult Mental Health and Suicidality
Source: JAMA Netw Open. 2023 Apr 14;6(4):e238415. doi: 10.1001/jamanetworkopen.2023.8415 (PMC10105313; doi:10.1001/jamanetworkopen.2023.8415)
Supplement: Supplement 1. — eTable 1. Alignment of Data From the National Survey on Drug Use and Health (NSDUH) With Years of State Supplemental Nutrition Assistance (SNAP) Eligibility Policies eTable 2. State Policy Adoption Status for Supplemental Nutrition Assistance Program (SNAP) Eligibility Policies, 2014-2017 eFigure 1. Associations of State Supplemental Nutrition Assistance Program (SNAP) Eligibility Policies With Past Year Mental Health Outcomes and Suicidality Among Adults Ages ≥18 Years eTable 3. Associations of State Supplemental Nutrition Assistance Program (SNAP) Eligibility Policies With Past Year Mental Health Outcomes and Suicidality Among Adults Ages ≥18 Years eFigure 2. Conceptual Diagram of Variables Likely to Be Operative in the Association Between State Adoption of Supplemental Nutrition Assistance (SNAP) Eligibility Policies and Mental Health and Suicidality Outcomes eFigure 3. Median Percentage of Adults Ages ≥18 Years With Past Year Major Depressive Episodes by Year and State Adoption of Supplemental Nutrition Assistance (SNAP) Eligibility Policies eFigure 4. Median Percentage of Adults Ages ≥18 Years With Past Year Mental Illness by Year and State Adoption of Supplemental Nutrition Assistance (SNAP) Eligibility Policies eFigure 5. Median Percentage of Adults Ages ≥18 Years With Past Year Serious Mental Illness by Year and State Adoption of Supplemental Nutrition Assistance (SNAP) Eligibility Policies eFigure 6. Median Percentage of Adults Ages ≥18 Years With Past Year Suicidal Ideation by Year and State Adoption of Supplemental Nutrition Assistance (SNAP) Eligibility Policies eFigure 7. Median Rate of Suicide Deaths Among Adults Ages ≥18 Years per 100 000 Population by Year and State Adoption of Supplemental Nutrition Assistance (SNAP) Eligibility Policies eTable 4. Adults Ages ≥18 Years With Past Year Mental Health Outcomes and Suicidality by State Adoption of Supplemental Nutrition Assistance (SNAP) Eligibility Policies eTable 5. Associations of State Supplemental N [file jamanetwopen-e238415-s001.pdf]

Supplemental Online Content

Austin AE, Frank M, Shanahan ME, Reyes HLM, Corbie G, Naumann RB. Association of state Supplemental Nutrition Assistance Program eligibility policies with adult mental health and suicidality. *JAMA Netw Open.* 2023;6(4):e238415. doi:10.1001/jamanetworkopen.2023.8415

eTable 1. Alignment of Data From the National Survey on Drug Use and Health (NSDUH) With Years of State Supplemental Nutrition Assistance (SNAP) Eligibility Policies

eTable 2. State Policy Adoption Status for Supplemental Nutrition Assistance Program (SNAP) Eligibility Policies, 2014-2017

eFigure 1. Associations of State Supplemental Nutrition Assistance Program (SNAP) Eligibility Policies With Past Year Mental Health Outcomes and Suicidality Among Adults Ages ≥18 Years

eTable 3. Associations of State Supplemental Nutrition Assistance Program (SNAP) Eligibility Policies With Past Year Mental Health Outcomes and Suicidality Among Adults Ages ≥18 Years

eFigure 2. Conceptual Diagram of Variables Likely to Be Operative in the Association Between State Adoption of Supplemental Nutrition Assistance (SNAP) Eligibility Policies and Mental Health and Suicidality Outcomes

eFigure 3. Median Percentage of Adults Ages ≥18 Years With Past Year Major Depressive Episodes by Year and State Adoption of Supplemental Nutrition Assistance (SNAP) Eligibility Policies

eFigure 4. Median Percentage of Adults Ages ≥18 Years With Past Year Mental Illness by Year and State Adoption of Supplemental Nutrition Assistance (SNAP) Eligibility Policies

eFigure 5. Median Percentage of Adults Ages ≥18 Years With Past Year Serious Mental Illness by Year and State Adoption of Supplemental Nutrition Assistance (SNAP) Eligibility Policies

eFigure 6. Median Percentage of Adults Ages ≥18 Years With Past Year Suicidal Ideation by Year and State Adoption of Supplemental Nutrition Assistance (SNAP) Eligibility Policies

eFigure 7. Median Rate of Suicide Deaths Among Adults Ages ≥18 Years per 100 000 Population by Year and State Adoption of Supplemental Nutrition Assistance (SNAP) Eligibility Policies

© 2023 Austin AE et al. *JAMA Network Open.*

eTable 4. Adults Ages ≥18 Years With Past Year Mental Health Outcomes and Suicidality by State Adoption of Supplemental Nutrition Assistance (SNAP) Eligibility Policies

eTable 5. Associations of State Supplemental Nutrition Assistance Program (SNAP) Eligibility Policies With Past Year Major Depressive Episode Among Adults

eTable 6. Associations of State Supplemental Nutrition Assistance Program (SNAP) Eligibility Policies With Past Year Mental Illness Among Adults

eTable 7. Associations of State Supplemental Nutrition Assistance Program (SNAP) Eligibility Policies With Suicide Deaths Among Adults

eTable 8. Associations of State Supplemental Nutrition Assistance Program (SNAP) Eligibility Policies With Past Year Suicidal Ideation Among Adults

eTable 9. Associations of State Supplemental Nutrition Assistance Program (SNAP) Eligibility Policies With Past Year Suicidal Ideation Among Adults

eFigure 8. Associations of State Supplemental Nutrition Assistance Program (SNAP) Eligibility Policies With Past Year Mental Health Symptoms and Disorders Among Adults Ages ≥18 Years Additionally Adjusted for Measures of the Mental Health Care Workforce

eTable 10. Associations of State Supplemental Nutrition Assistance Program (SNAP) Eligibility Policies With Past Year Mental Health Symptoms and Disorders Among Adults Ages ≥18 Years Additionally Adjusted for Measures of the Mental Health Care Workforce

eFigure 9. Associations of State Supplemental Nutrition Assistance Program (SNAP) Eligibility Policies With Unintentional Motor Vehicle Deaths Among Adults Ages ≥18 Years

eTable 11. Associations of State Supplemental Nutrition Assistance Program (SNAP) Eligibility Policies With Unintentional Motor Vehicle Deaths Among Adults Ages ≥18 Years

This supplemental material has been provided by the authors to give readers additional information about their work..

© 2023 Austin AE et al. *JAMA Network Open*.

eTable 1. Alignment of data from the National Survey on Drug Use and Health (NSDUH) with years of state Supplemental Nutrition Assistance (SNAP) eligibility policies

State-level estimates of past year mental health outcomes from NSDUH	Year of SNAP eligibility policy adoption
2015-2016	2014
2016-2017	2015
2017-2018	2016
2018-2019	2017

Because of the retrospective nature of the mental health outcomes captured by the National Survey on Drug Use and Health (NSDUH; e.g., major depressive episode in the past year), we created a 1-year lag between state adoption of Supplemental Nutrition Assistance Program (SNAP) eligibility policies and the first year of the 2-year state-level average of the number of adults with each outcome. For example, we considered an adult included in the 2015-2016 NSDUH state-level estimates to have been exposed to the SNAP eligibility policies present in 2014. This ensured that the mental health outcomes occurred after states had adopted the SNAP eligibility policies.

© 2023 Austin AE et al. *JAMA Network Open.*

eTable 2. State policy adoption status for Supplemental Nutrition Assistance Program (SNAP) eligibility policies, 2014-2017

State	Year	Eliminated the asset test	Increased income limit	Both policies
AK	2014			
AK	2015			
AK	2016			
AK	2017			
AL	2014	x		
AL	2015	x		
AL	2016	x		
AL	2017	x		
AR	2014			
AR	2015			
AR	2016			
AR	2017			
AZ	2014			x
AZ	2015			x
AZ	2016			x
AZ	2017			x
CA	2014	x		
CA	2015			x
CA	2016			x
CA	2017			x
CO	2014	x		
CO	2015	x		
CO	2016	x		
CO	2017	x		
CT	2014			x
CT	2015			x
CT	2016			x
CT	2017			x
DC	2014			x
DC	2015			x
DC	2016			x
DC	2017			x
DE	2014			x
DE	2015			x
DE	2016			x
DE	2017			x
FL	2014			x
FL	2015			x

© 2023 Austin AE et al. *JAMA Network Open.*

FL	2016			x
FL	2017			x
GA	2014	x		
GA	2015	x		
GA	2016	x		
GA	2017	x		
HI	2014			x
HI	2015			x
HI	2016			x
HI	2017			x
IA	2014			x
IA	2015			x
IA	2016			x
IA	2017			x
ID	2014			
ID	2015			
ID	2016			
ID	2017			
IL	2014	x		
IL	2015	x		
IL	2016			x
IL	2017			x
IN	2014			
IN	2015			
IN	2016			
IN	2017			
KS	2014			
KS	2015			
KS	2016			
KS	2017			
KY	2014	x		
KY	2015	x		
KY	2016	x		
KY	2017	x		
LA	2014	x		
LA	2015			
LA	2016			
LA	2017			
MA	2014	x		
MA	2015	x		
MA	2016			x
MA	2017			x

© 2023 Austin AE et al. *JAMA Network Open.*

MD	2014			x
MD	2015			x
MD	2016			x
MD	2017			x
ME	2014			x
ME	2015			x
ME	2016			x
ME	2017			x
MI	2014		x	
MI	2015		x	
MI	2016		x	
MI	2017		x	
MN	2014			x
MN	2015			x
MN	2016			x
MN	2017			x
MO	2014			
MO	2015			
MO	2016			
MO	2017			
MS	2014	x		
MS	2015	x		
MS	2016	x		
MS	2017	x		
MT	2014			x
MT	2015			x
MT	2016			x
MT	2017			x
NC	2014			x
NC	2015			x
NC	2016			x
NC	2017			x
ND	2014			x
ND	2015			x
ND	2016			x
ND	2017			x
NE	2014			
NE	2015			
NE	2016			
NE	2017			
NH	2014			x
NH	2015			x

© 2023 Austin AE et al. *JAMA Network Open.*

NH	2016			x
NH	2017			x
NJ	2014			x
NJ	2015			x
NJ	2016			x
NJ	2017			x
NM	2014			x
NM	2015			x
NM	2016			x
NM	2017			x
NV	2014			x
NV	2015			x
NV	2016			x
NV	2017			x
NY	2014			x
NY	2015			x
NY	2016			x
NY	2017			x
OH	2014	x		
OH	2015	x		
OH	2016	x		
OH	2017	x		
OK	2014	x		
OK	2015	x		
OK	2016	x		
OK	2017	x		
OR	2014			x
OR	2015			x
OR	2016			x
OR	2017			x
PA	2014		x	
PA	2015			x
PA	2016			x
PA	2017			x
RI	2014			x
RI	2015			x
RI	2016			x
RI	2017			x
SC	2014	x		
SC	2015	x		
SC	2016	x		
SC	2017	x		

© 2023 Austin AE et al. *JAMA Network Open.*

SD	2014			
SD	2015			
SD	2016			
SD	2017			
TN	2014			
TN	2015			
TN	2016			
TN	2017			
TX	2014		x	
TX	2015		x	
TX	2016		x	
TX	2017		x	
UT	2014			
UT	2015			
UT	2016			
UT	2017			
VA	2014			
VA	2015			
VA	2016			
VA	2017			
VT	2014			x
VT	2015			x
VT	2016			x
VT	2017			x
WA	2014			x
WA	2015			x
WA	2016			x
WA	2017			x
WI	2014			x
WI	2015			x
WI	2016			x
WI	2017			x
WV	2014	x		
WV	2015	x		
WV	2016	x		
WV	2017	x		
WY	2014			
WY	2015			
WY	2016			
WY	2017			

Note: In analyses, we excluded state-years with an increased income limit only from analyses due to the small number and percentage (N=9, 4.4%).

© 2023 Austin AE et al. *JAMA Network Open.*

eFigure 1. Associations of state Supplemental Nutrition Assistance Program (SNAP) eligibility policies with past year mental health outcomes and suicidality among adults ages ≥18 years (n=204 state-years)

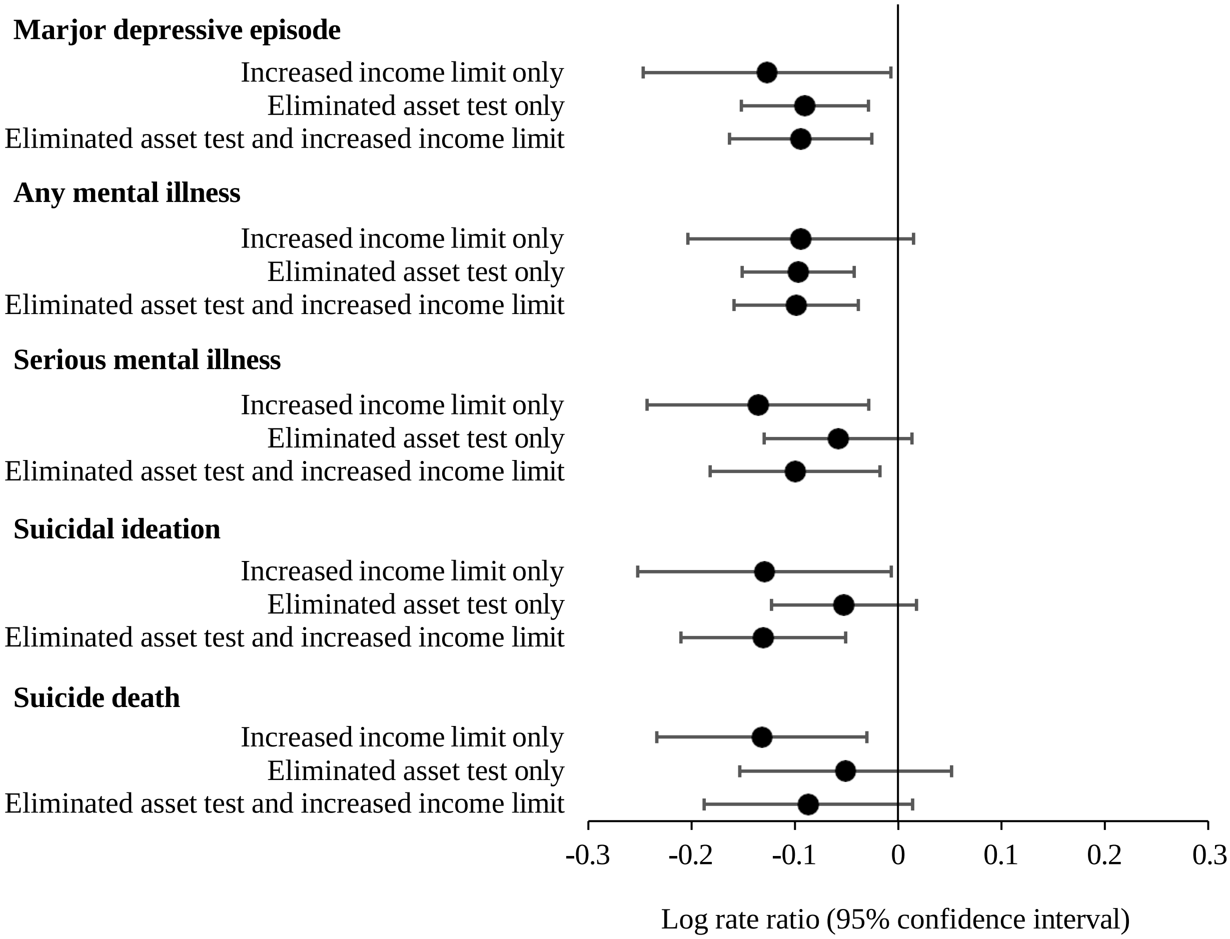

[a]Adjusted for a linear time trend and state minimum wage, refundable Earned Income Tax Credit, maximum Temporary Assistance for Needy Families benefit for a family of 3, Medicaid expansion, recreational marijuana legalization, percent population unemployed, and median household income
Note: All 50 states and the District of Columbia included in analyses.

© 2023 Austin AE et al. *JAMA Network Open.*

eTable 3. Associations of state Supplemental Nutrition Assistance Program (SNAP) eligibility policies with past year mental health outcomes and suicidality among adults ages ≥18 years (n=204 state-years)

	Adjusted[a] rate ratio (RR)	95% confidence interval (CI)
**Major depressive episode**		
Neither policy	1.00	
Eliminated asset test only	0.91	0.86, 0.97
Increased income limit only	0.88	0.78, 0.99
Eliminated the asset test and increased the income limit	0.91	0.85, 0.97
**Any mental illness**		
Neither policy	1.00	
Eliminated asset test only	0.91	0.86, 0.96
Increased income limit only	0.91	0.82, 1.02
Eliminated the asset test and increased the income limit	0.91	0.85, 0.96
**Serious mental illness**		
Neither policy	1.00	
Eliminated asset test only	0.94	0.88, 1.01
Increased income limit only	0.87	0.78, 0.97
Eliminated the asset test and increased the income limit	0.91	0.83, 0.98
**Suicidal ideation**		
Neither policy	1.00	
Eliminated asset test only	0.95	0.88, 1.02
Increased income limit only	0.88	0.78, 0.99
Eliminated the asset test and increased the income limit	0.88	0.81, 0.95
**Suicide deaths**		
Neither policy	1.00	
Eliminated asset test only	0.95	0.86, 1.05
Increased income limit only	0.88	0.79, 0.97
Eliminated the asset test and increased the income limit	0.92	0.83, 1.01

[a]Adjusted for a linear time trend and state minimum wage, refundable Earned Income Tax Credit, maximum Temporary Assistance for Needy Families benefit for a family of 3, Medicaid expansion, recreational marijuana legalization, percent population unemployed, and median household income
Note: All 50 states and the District of Columbia included in analyses

© 2023 Austin AE et al. *JAMA Network Open.*

eFigure 2. Conceptual diagram of variables likely to be operative in the association between state adoption of Supplemental Nutrition Assistance (SNAP) eligibility policies and mental health and suicidality outcomes

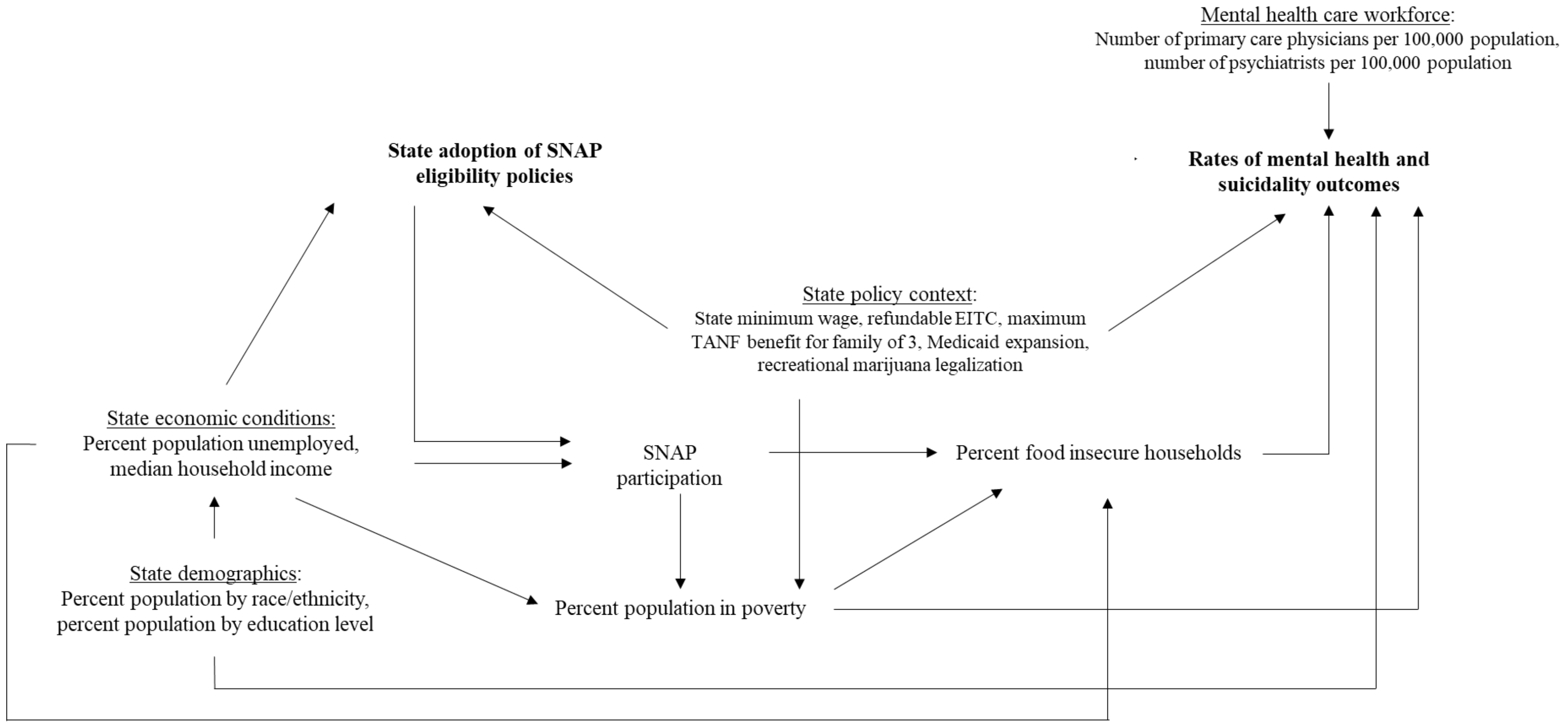

© 2023 Austin AE et al. *JAMA Network Open.*

eFigure 3. Median percent of adults ages ≥18 years with past year major depressive episodes by year and state adoption of Supplemental Nutrition Assistance (SNAP) eligibility policies (N=195 state-years)

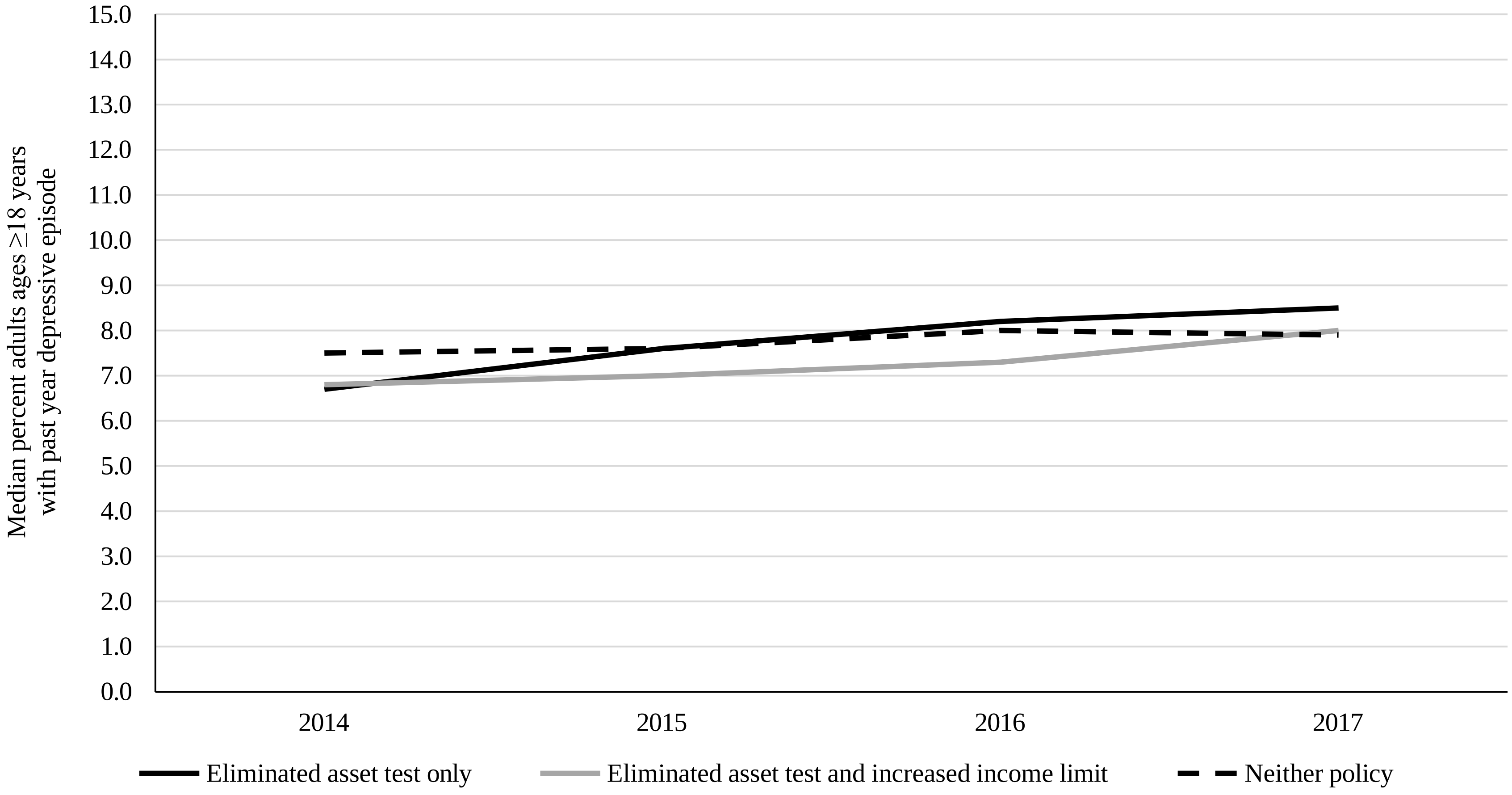

Note: Data from the National Survey on Drug Use and Health (NSDUH) State-Level Small Area Estimates for 2015-2016, 2016-2017, 2017-2018, and 2018- 2019. Because all data are retrospectively reported in NSDUH, we created a 1-year lag between state adoption of the SNAP eligibility policies and the first year of the 2-year state-level averages for our outcomes of interest. For example, we considered the 2015-2016 NSDUH state estimates to align with SNAP eligibility policies in 2014.

© 2023 Austin AE et al. *JAMA Network Open.*

eFigure 4. Median percent of adults ages ≥18 years with past year mental illness by year and state adoption of Supplemental Nutrition Assistance (SNAP) eligibility policies (N=195 state-years)

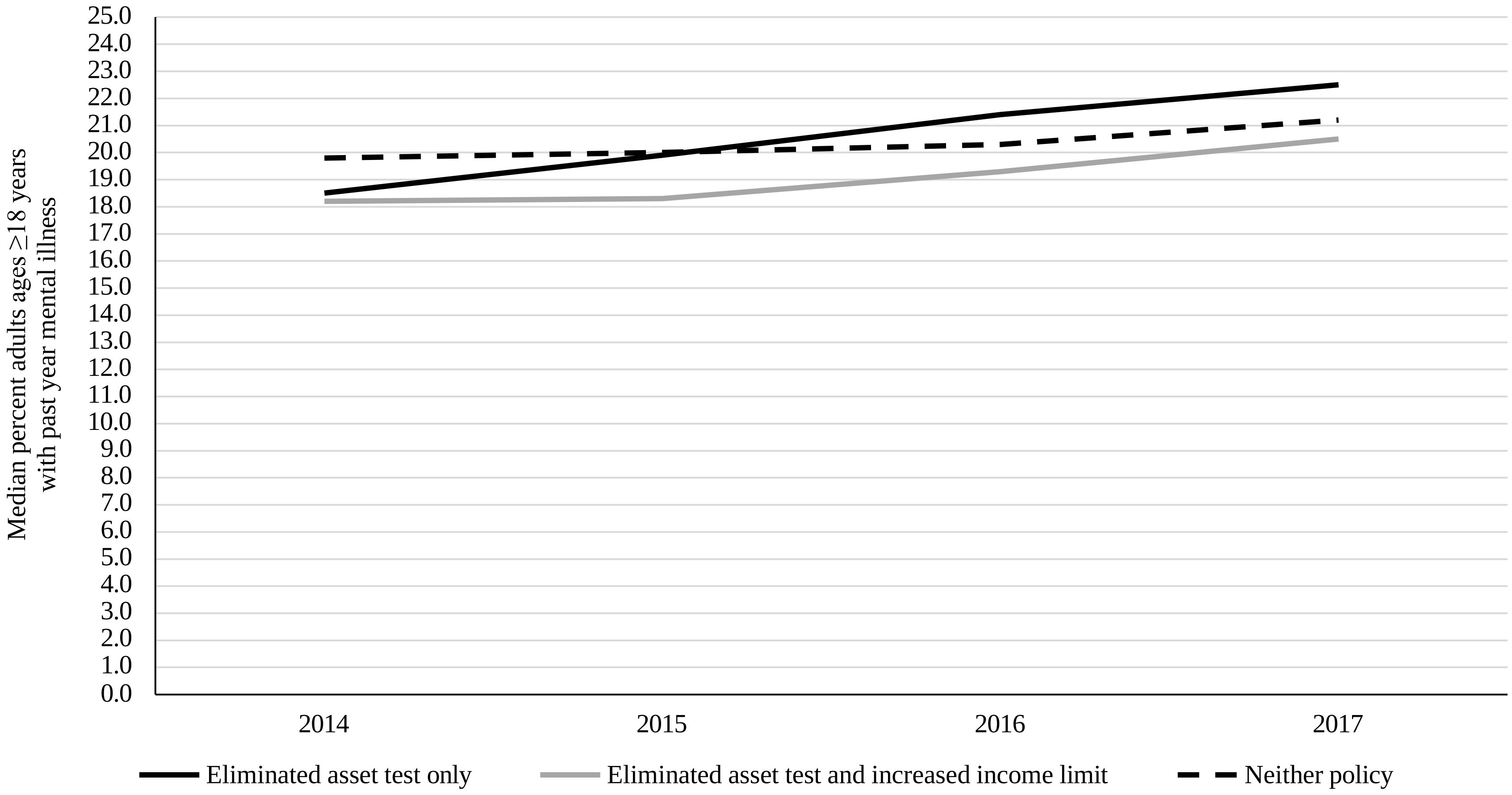

Note: Data from the National Survey on Drug Use and Health (NSDUH) State-Level Small Area Estimates for 2015-2016, 2016-2017, 2017-2018, and 2018- 2019. Because all data are retrospectively reported in NSDUH, we created a 1-year lag between state adoption of the SNAP eligibility policies and the first year of the 2-year state-level averages for our outcomes of interest. For example, we considered the 2015-2016 NSDUH state estimates to align with SNAP eligibility policies in 2014.

© 2023 Austin AE et al. *JAMA Network Open.*

eFigure 5. Median percent of adults ages ≥18 years with past year serious mental illness by year and state adoption of Supplemental Nutrition Assistance (SNAP) eligibility policies (N=195 state-years)

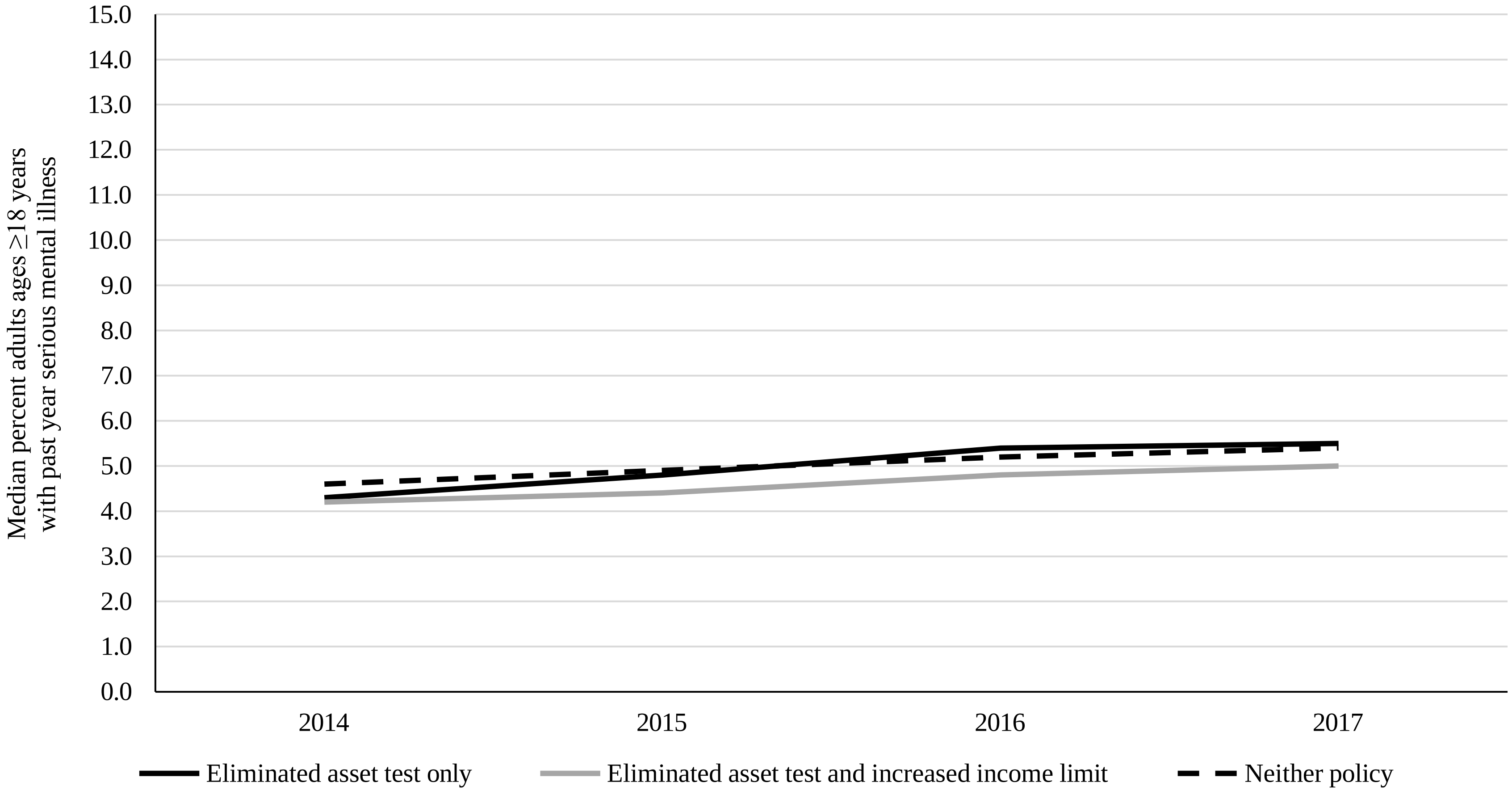

Note: Data from the National Survey on Drug Use and Health (NSDUH) State-Level Small Area Estimates for 2015-2016, 2016-2017, 2017-2018, and 2018- 2019. Because all data are retrospectively reported in NSDUH, we created a 1-year lag between state adoption of the SNAP eligibility policies and the first year of the 2-year state-level averages for our outcomes of interest. For example, we considered the 2015-2016 NSDUH state estimates to align with SNAP eligibility policies in 2014.

© 2023 Austin AE et al. *JAMA Network Open.*

eFigure 6. Median percent of adults ages ≥18 years with past year suicidal ideation by year and state adoption of Supplemental Nutrition Assistance (SNAP) eligibility policies (N=195 state-years)

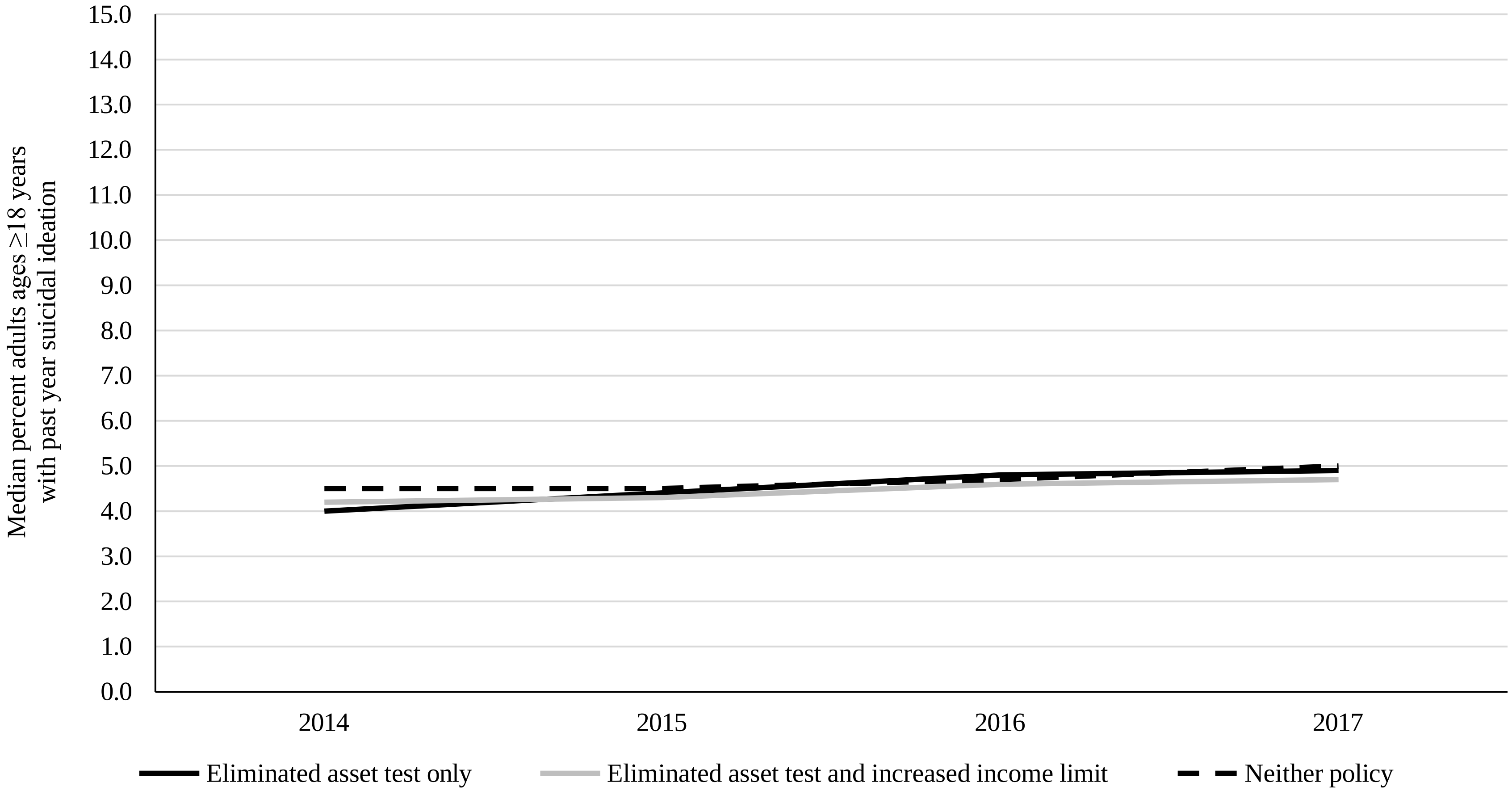

Note: Data from the National Survey on Drug Use and Health (NSDUH) State-Level Small Area Estimates for 2015-2016, 2016-2017, 2017-2018, and 2018- 2019. Because all data are retrospectively reported in NSDUH, we created a 1-year lag between state adoption of the SNAP eligibility policies and the first year of the 2-year state-level averages for our outcomes of interest. For example, we considered the 2015-2016 NSDUH state estimates to align with SNAP eligibility policies in 2014.

© 2023 Austin AE et al. *JAMA Network Open.*

eFigure 7. Median rate of suicide deaths among adults ages ≥18 years per 100,000 population by year and state adoption of Supplemental Nutrition Assistance (SNAP) eligibility policies (N=195 state-years)

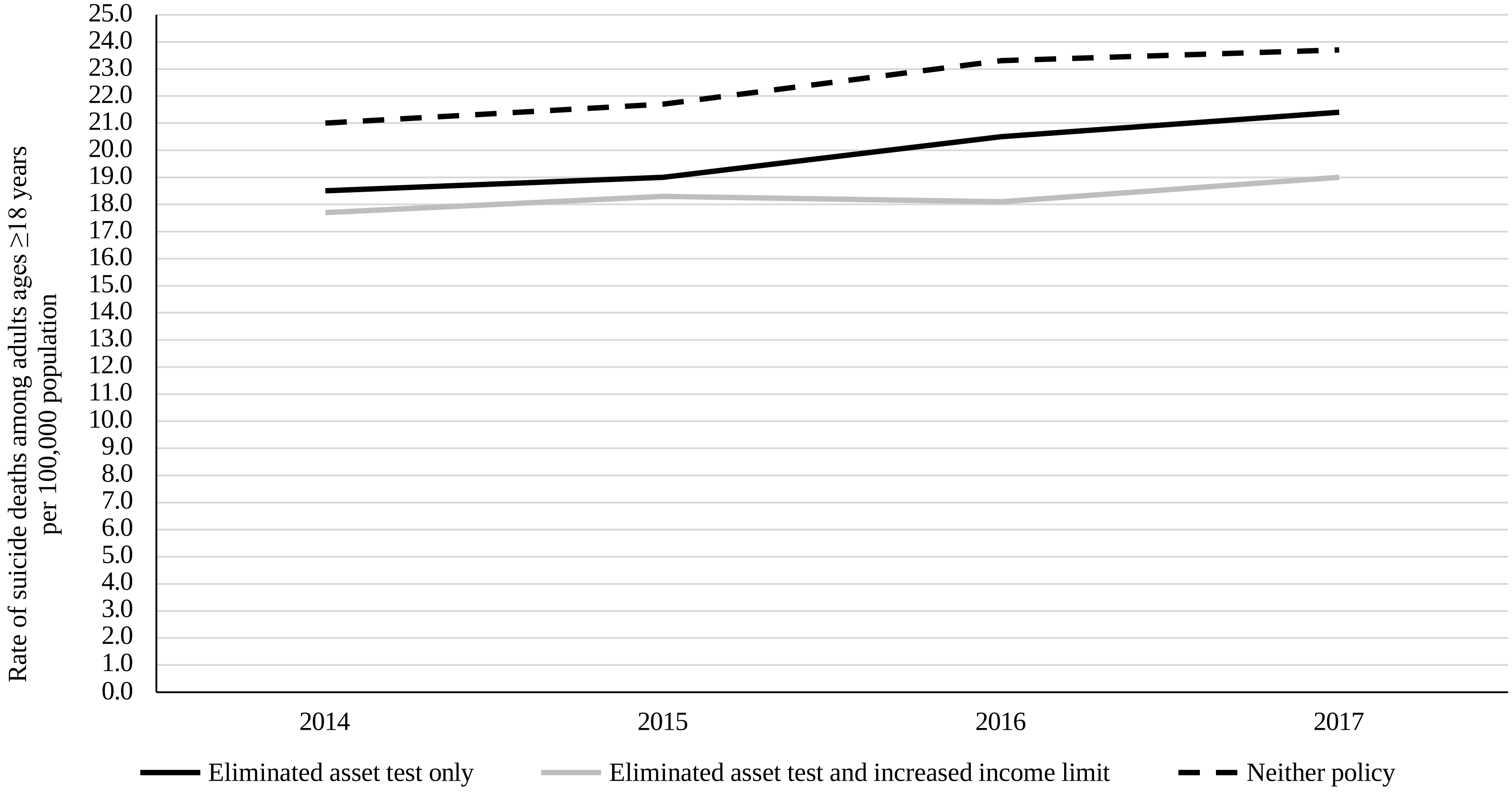

Note: Data from the National Vital Statistics System (NVSS) indicating causes of death for U.S. residents based on death certificates.

© 2023 Austin AE et al. *JAMA Network Open*.

eTable 4. Adults ages ≥18 years with past year mental health outcomes and suicidality by state adoption of Supplemental Nutrition Assistance (SNAP) eligibility policies (N=195 state-years)

	Percent with a major depressive episode	Percent with any mental illness	Percent with a serious mental illness	Percent with suicidal ideation	Number of suicide deaths per 100,000 population
	25th percentile, median, 75th percentile	25th percentile, median, 75th percentile	25th percentile, median, 75th percentile	25th percentile, median, 75th percentile	25th percentile, median, 75th percentile
**Eliminated the asset test only** (n=42 state-years)	6.9, 7.6, 8.3	18.3, 19.9, 21.5	4.4, 5.0, 5.5	4.0, 4.6, 4.9	17.3, 19.5, 23.5
**Eliminated the asset test and increased the income limit** (n=102 state-years)	6.7, 7.3, 8.1	18.0, 19.1, 20.6	4.2, 4.6, 5.2	4.0, 4.5, 4.9	13.8, 18.5, 22.9
**Neither policy** (n=51 state-years)	7.1, 7.7, 8.2	18.8, 20.3, 21.5	4.6, 5.2, 5.4	4.4, 4.7, 5.2	19.5, 22.6, 26.9

© 2023 Austin AE et al. *JAMA Network Open*.

eTable 5. Associations of state Supplemental Nutrition Assistance Program (SNAP) eligibility policies with past year major depressive episode among adults (n=195 state-years)

	Adjusted rate ratio (RR) and 95% confidence interval (CI)
**Exposures**	
Neither policy	1.00
Eliminated the asset test only	0.92 (0.87, 0.98)
Eliminated the asset test and increased the income limit	0.92 (0.86, 0.99)
**Covariates**	
State minimum wage	0.99 (0.96, 1.02)
Refundable Earned Income Tax Credit	
No	1.00
Yes	0.99 (0.94, 1.04)
Maximum Temporary Assistance for Needy Families benefit for family of 3	1.00 (0.99, 1.00)
Medicaid expansion	
No	1.00
Yes	1.04 (0.99, 1.10)
Recreational marijuana legalization	
No	
Yes	1.00
Percent population unemployed	1.06 (1.00, 1.13)
Median household income	0.99 (0.98, 1.01)
Linear time trend	1.04 (1.02, 1.05)

© 2023 Austin AE et al. *JAMA Network Open*.

eTable 6. Associations of state Supplemental Nutrition Assistance Program (SNAP) eligibility policies with past year mental illness among adults (n=195 state-years)

	Adjusted rate ratio (RR) and 95% confidence interval (CI)
**Exposures**	
Neither policy	1.00
Eliminated the asset test only	0.91(0.87, 0.97)
Eliminated the asset test and increased the income limit	0.92 (0.87, 0.98)
**Covariates**	
State minimum wage	1.00 (0.98, 1.02)
Refundable Earned Income Tax Credit	
No	1.00
Yes	0.96 (0.93, 1.00)
Maximum Temporary Assistance for Needy Families benefit for family of 3	1.00 (0.99, 1.00)
Medicaid expansion	
No	1,00
Yes	1.04 (1.00, 1.08)
Recreational marijuana legalization	
No	1.00
Yes	1.06 (1.01, 1.10)
Percent population unemployed	1.00 (0.98, 1.02)
Median household income	1.00 (1.00, 1.00)
Linear time trend	1.03 (1.01, 1.05)

© 2023 Austin AE et al. *JAMA Network Open*.

eTable 7. Associations of state Supplemental Nutrition Assistance Program (SNAP) eligibility policies with suicide deaths among adults (n=195 state-years)

	Adjusted rate ratio (RR) and 95% confidence interval (CI)
**Exposures**	
Neither policy	1.00
Eliminated the asset test only	0.96 (0.87, 1.06)
Eliminated the asset test and increased the income limit	0.93 (0.84, 1.02)
**Covariates**	
State minimum wage	1.00 (0.96, 1.03)
Refundable Earned Income Tax Credit	
No	1.00
Yes	0.97 (0.93, 1.01)
Maximum Temporary Assistance for Needy Families benefit for family of 3	0.99 (0.99, 1.00)
Medicaid expansion	
No	1.00
Yes	1.03 (0.97, 1.11)
Recreational marijuana legalization	
No	1.00
Yes	1.09 (1.03, 1.16)
Percent population unemployed	0.98 (0.95, 1.00)
Median household income	1.00 (0.99, 1.00)
Linear time trend	1.05 (1.03, 1.07)

© 2023 Austin AE et al. *JAMA Network Open*.

eTable 8. Associations of state Supplemental Nutrition Assistance Program (SNAP) eligibility policies with past year suicidal ideation among adults (n=195 state-years)

	Adjusted rate ratio (RR) and 95% confidence interval (CI)
**Exposures**	
Neither policy	1.00
Eliminated the asset test only	0.96 (0.89, 1.03)
Eliminated the asset test and increased the income limit	0.89 (0.82, 0.96)
**Covariates**	
State minimum wage	0.99 (0.96, 1.02)
Refundable Earned Income Tax Credit	
No	1.00
Yes	0.95 (0.91, .099)
Maximum Temporary Assistance for Needy Families benefit for family of 3	1.00 (0.99, 1.00)
Medicaid expansion	
No	1.00
Yes	1.07 (1.01, 1.14)
Recreational marijuana legalization	
No	1.00
Yes	1.09 (1.02, 1.17)
Percent population unemployed	0.97 (0.94, 1.00)
Median household income	1.00 (1.00, 1.01)
Linear time trend	1.03 (1.00, 1.06)

© 2023 Austin AE et al. *JAMA Network Open*.

eTable 9. Associations of state Supplemental Nutrition Assistance Program (SNAP) eligibility policies with past year suicidal ideation among adults (n=195 state-years)

	Adjusted rate ratio (RR) and 95% confidence interval (CI)
**Exposures**	
Neither policy	1.00
Eliminated the asset test only	0.96 (0.89, 1.03)
Eliminated the asset test and increased the income limit	0.89 (0.82, 0.96)
**Covariates**	
State minimum wage	1.00 (0.97, 1.02)
Refundable Earned Income Tax Credit	
No	1.00
Yes	0.96 (0.90, 1.02)
Maximum Temporary Assistance for Needy Families benefit for family of 3	0.99 (0.99, 1.00)
Medicaid expansion	
No	1.00
Yes	0.99 (0.95, 1.03)
Recreational marijuana legalization	
No	1.00
Yes	1.05 (0.99, 1.11)
Percent population unemployed	1.00 (0.98, 1.01)
Median household income	1.00 (1.00, 1.01)
Linear time trend	1.03 (1.02, 1.05)

© 2023 Austin AE et al. *JAMA Network Open*.

eFigure 8. Associations of state Supplemental Nutrition Assistance Program (SNAP) eligibility policies with past year mental health symptoms and disorders among adults ages ≥18 years additionally adjusted for measures of the mental health care workforce (N=195 state-years)

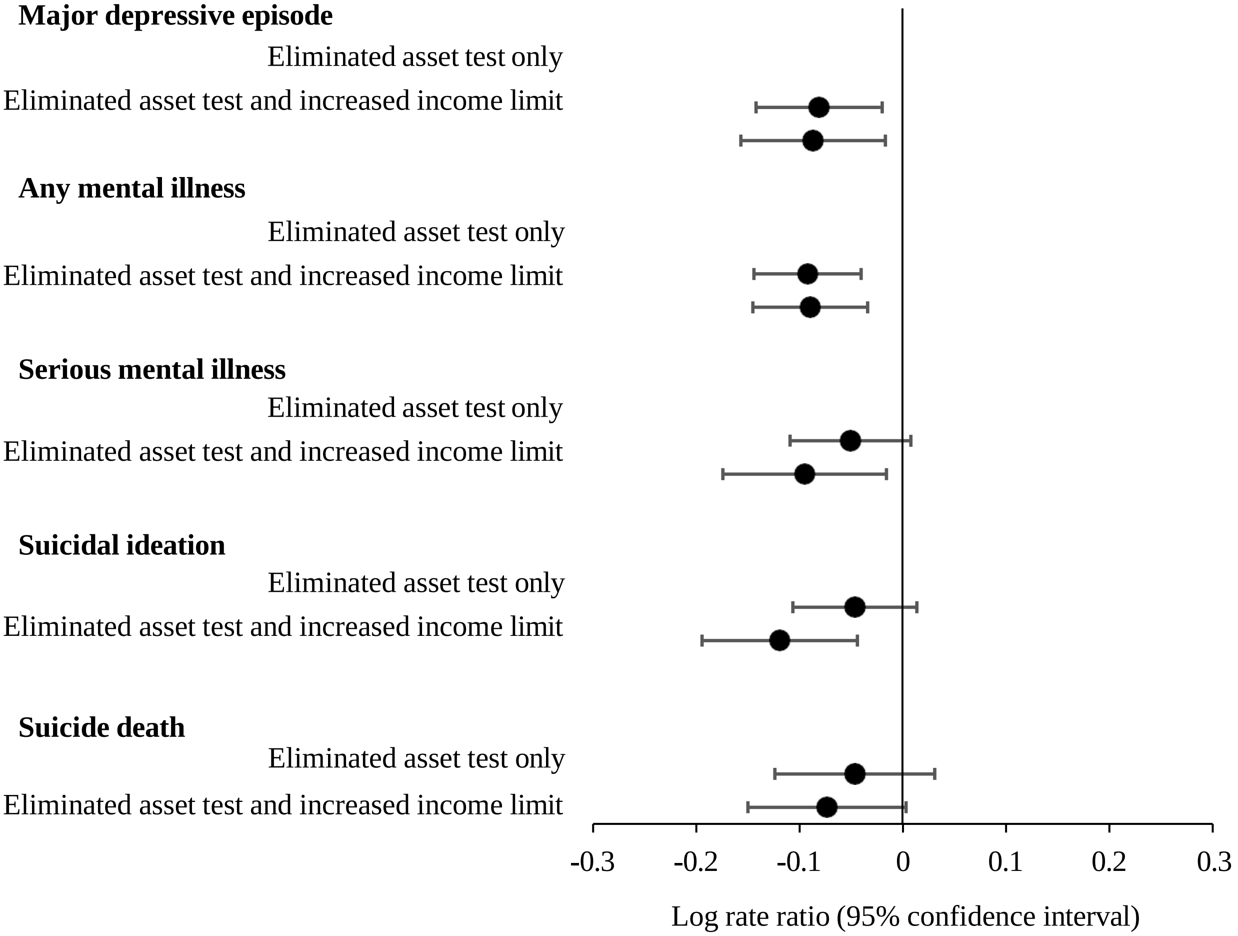

[a]Adjusted for a linear time trend and state minimum wage, refundable Earned Income Tax Credit, maximum Temporary Assistance for Needy Families benefit for a family of 3, Medicaid expansion, recreational marijuana legalization, percent population unemployed, median household income, number of psychiatrists per 100,000 population, and number of primary care providers per 100,000 population
Note: Comparison group is state-years that did not have the asset test eliminated or the income limit increased for SNAP eligibility.

© 2023 Austin AE et al. *JAMA Network Open.*

eTable 10. Associations of state Supplemental Nutrition Assistance Program (SNAP) eligibility policies with past year mental health symptoms and disorders among adults ages ≥18 years additionally adjusted for measures of the mental health care workforce (N=195 state-years)

	Adjusted[a] rate ratio (RR)	95% confidence interval (CI)
**Major depressive episode**		
Neither policy	1.00	
Eliminated asset test only	0.92	0.87, 0.98
Eliminated the asset test and increased the income limit	0.92	0.86, 0.98
**Any mental illness**		
Neither policy	1.00	
Eliminated asset test only	0.91	0.86, 0.96
Eliminated the asset test and increased the income limit	0.91	0.86, 0.97
**Serious mental illness**		
Neither policy	1.00	
Eliminated asset test only	0.95	0.90, 1.01
Eliminated the asset test and increased the income limit	0.91	0.84, 0.98
**Suicidal ideation**		
Neither policy	1.00	
Eliminated asset test only	0.95	0.90, 1.01
Eliminated the asset test and increased the income limit	0.89	0.82, 0.96
**Suicide death**		
Neither policy	1.00	
Eliminated asset test only	0.95	0.88, 1.03
Eliminated the asset test and increased the income limit	0.93	0.86, 1.00

[a]Adjusted for a linear time trend and state minimum wage, refundable Earned Income Tax Credit, maximum Temporary Assistance for Needy Families benefit for a family of 3, Medicaid expansion, recreational marijuana legalization, percent population unemployed, median household income, number of psychiatrists per 100,000 population, and number of primary care providers per 100,000 population

© 2023 Austin AE et al. *JAMA Network Open*.

eFigure 9. Associations of state Supplemental Nutrition Assistance Program (SNAP) eligibility policies with unintentional motor vehicle deaths among adults ages ≥18 years (N=195 state-years)

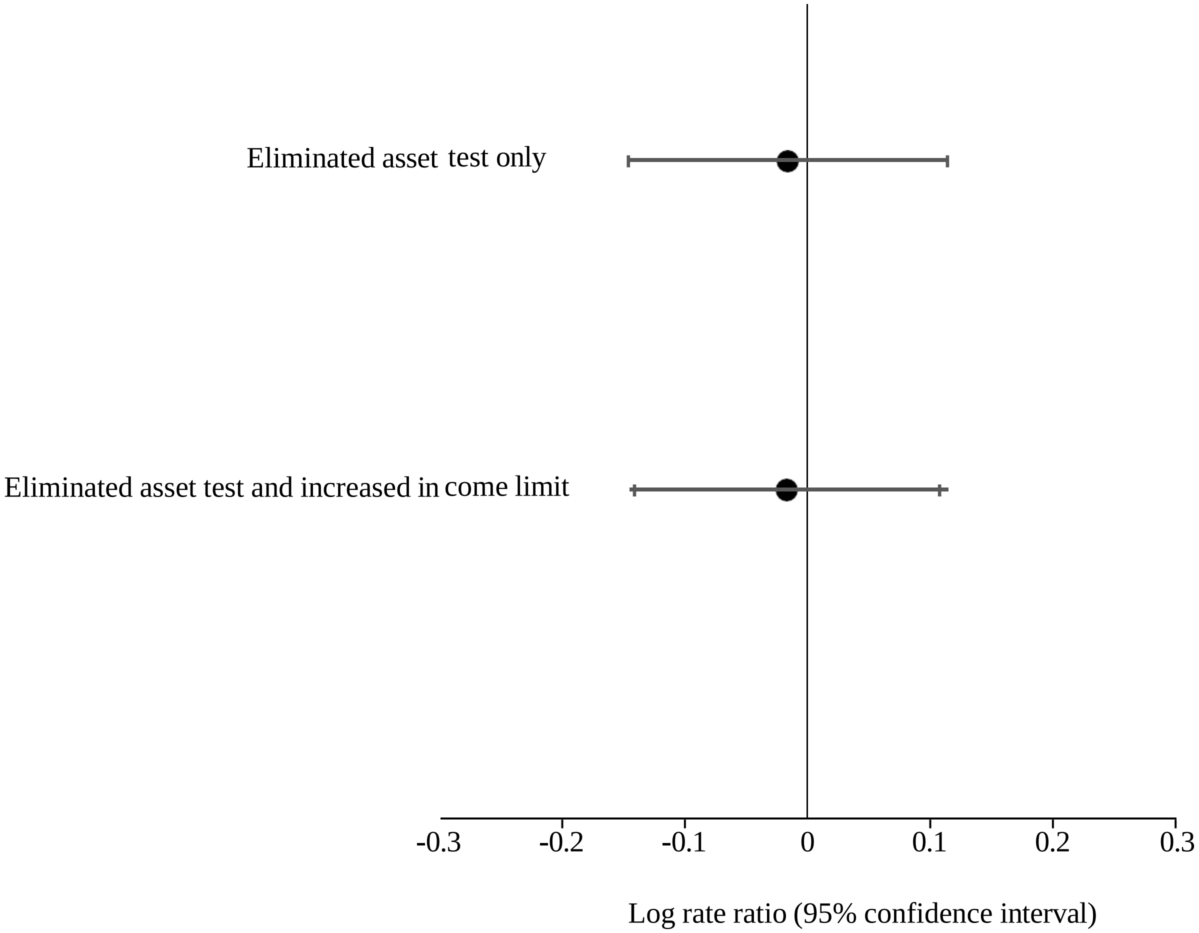

[a]Adjusted for a linear time trend. Note: Comparison group is state-years that did not have the asset test eliminated or the income limit increased for SNAP eligibility.

© 2023 Austin AE et al. *JAMA Network Open*.

eTable 11. Associations of state Supplemental Nutrition Assistance Program (SNAP) eligibility policies with unintentional motor vehicle deaths among adults ages ≥18 years (N=195 state-years)

	Adjusted[a] rate ratio (RR)	95% confidence interval (CI)
**Unintentional motor vehicle deaths**		
Neither policy	1.00	
Eliminated asset test only	0.98	0.86, 1.12
Eliminated the asset test and increased the income limit	0.98	0.87, 1.11

[a]Adjusted for a linear time trend

© 2023 Austin AE et al. *JAMA Network Open*.
