# Supplementary material for: Association of State Supplemental Nutrition Assistance Program Eligibility Policies With Adult Mental Health and Suicidality
Source: JAMA Netw Open. 2023 Apr 14;6(4):e238415. doi: 10.1001/jamanetworkopen.2023.8415 (PMC10105313; doi:10.1001/jamanetworkopen.2023.8415)
Supplement: Supplement 2. — Data Sharing Statement [file jamanetwopen-e238415-s002.pdf]

Data Sharing Statement

Austin. Association of State Supplemental Nutrition Assistance Program (SNAP) Eligibility Policies With Adult Mental Health and Suicidality. *JAMA Netw Open*. Published April 14, 2023. doi:10.1001/jamanetworkopen.2023.8415

Data
Data available: No

Additional Information
Explanation for why data not available: Data are publicly available online; websites where data can be obtained are provided in the reference section.
